# Glucosinolate Abundance and Composition in Brassicaceae Influence Sequestration in a Specialist Flea Beetle

**DOI:** 10.1007/s10886-020-01144-y

**Published:** 2020-01-17

**Authors:** Zhi-Ling Yang, Grit Kunert, Theresa Sporer, Johannes Körnig, Franziska Beran

**Affiliations:** 1 Research Group Sequestration and Detoxification in Insects, Max Planck Institute for Chemical Ecology, Hans-Knoell-Strasse 8, D-07745 Jena, Germany; 2 Department of Biochemistry, Max Planck Institute for Chemical Ecology, Hans-Knoell-Strasse 8, D-07745 Jena, Germany

**Keywords:** Plant-insect interaction, *Phyllotreta*, Sequestration, Glucosinolate, Excretion, Metabolism, Adaptation

## Abstract

The horseradish flea beetle *Phyllotreta armoraciae* exclusively feeds on Brassicaceae, which contain glucosinolates as characteristic defense compounds. Although glucosinolates are usually degraded by plant enzymes (myrosinases) to toxic isothiocyanates after ingestion, *P. armoraciae* beetles sequester glucosinolates. Between and within brassicaceous plants, the glucosinolate content and composition can differ drastically. But how do these factors influence sequestration in *P. armoraciae*? To address this question, we performed a five-day feeding experiment with three *Arabidopsis thaliana* lines that differ four-fold in glucosinolate content and the composition of aliphatic and indolic glucosinolates. We quantified the amounts of ingested, sequestered, and excreted glucosinolates, and analyzed the changes in glucosinolate levels and composition in beetles before and after feeding on *Arabidopsis*. *P. armoraciae* accumulated almost all ingested glucosinolate types. However, some glucosinolates were accumulated more efficiently than others, and selected glucosinolates were modified by the beetles. The uptake of new glucosinolates correlated with a decrease in the level of stored glucosinolates so that the total glucosinolate content remained stable at around 35 nmol/mg beetle fresh weight. Beetles excreted previously stored as well as ingested glucosinolates from *Arabidopsis*, which suggests that *P. armoraciae* regulate their endogenous glucosinolate level by excretion. The metabolic fate of ingested glucosinolates, i.e. the proportions of sequestered and excreted glucosinolates, depended on glucosinolate type, content, and composition in the food plant. Overall, *P. armoraciae* sequestered and excreted up to 41% and 31% of the total ingested aliphatic and indolic glucosinolates from *Arabidopsis*, respectively. In summary, we show that glucosinolate variability in Brassicaceae influences the composition but not the level of sequestered glucosinolates in *P. armoraciae* beetles.

**Electronic supplementary material:**

The online version of this article (10.1007/s10886-020-01144-y) contains supplementary material, which is available to authorized users.

## Introduction

Many plants utilize two-component defenses that are activated upon tissue damage. These defense systems consist of glucosylated secondary metabolites, e.g. cyanogenic, iridoid, and benzoxazinoid glucosides that are separately stored from activating β-glucosidases in intact plant tissue (Pentzold et al. 2014b). A well-studied activated defense is the glucosinolate (GLS)-myrosinase system in plants of the order Brassicales, also known as the “mustard oil bomb” (Halkier and Gershenzon 2006; Lüthy and Matile 1984). When leaf damage disrupts the spatial separation between GLS and myrosinase, GLS are hydrolyzed to unstable aglucones that are further converted to breakdown products including highly reactive isothiocyanates (Wittstock et al. 2016). To date, more than 130 different GLS have been identified in plants, and have been broadly classified according to the structure of their amino acid-derived side chain as benzenic, indolic, or aliphatic GLS (Agerbirk and Olsen 2012).

Some herbivorous insects also possess activated defense systems that consist of glucosylated compounds, either sequestered from food plants or synthesized *de novo,* and insect-derived activating β-glucosidase enzymes (Beran et al. 2019). For example, larvae of the six spot burnet moth, *Zygaena filipendulae* (L.) (Zygaenidae), sequester and *de novo* synthesize cyanogenic glucosides, and produce a cyanogenic β-glucosidase (Jensen et al. 2011; Zagrobelny et al. 2018). To sequester cyanogenic glucosides from their food plant, *Z. filipendulae* larvae prevent hydrolysis by plant cyanogenic *β*-glucosidases. For instance, reduced plant *β*-glucosidase activity under the alkaline gut pH conditions might facilitate the sequestration of ingested cyanogenic glucosides by *Z. filipendulae* larvae (Pentzold et al. 2014a). Another mechanism that allows insects to sequester glucosylated defense compounds is by absorbing them across the gut epithelium before they are activated in the gut lumen. This strategy was suggested to prevent the hydrolysis of ingested GLS in larvae of the turnip sawfly, *Athalia rosae* (L.) (Tenthrenidinae), although surprisingly, the sequestered GLS cannot be activated by the insect for its defense (Abdalsamee et al. 2014; Müller and Wittstock 2005). Thus, sequestration of glucosylated plant defense compounds might function as a detoxification strategy by preventing the formation of toxic breakdown products (Pentzold et al. 2014b) as an alternative to serving a defensive function.

We previously discovered that adults of the striped flea beetle, *Phyllotreta striolata* (Fabricius) (Chrysomelidae), have a high capacity to sequester certain aliphatic GLS from their brassicaceous food plants, and that the insects produce myrosinase that converts sequestered GLS to toxic isothiocyanates (Beran et al. 2014). *P. striolata* is an oligophagous species that feeds on many different cultivated and wild Brassicaceae plant species and thus encounters wide ranges of GLS concentrations and compositions in its food plants (Gikonyo et al. 2019). Analyses of the GLS sequestration patterns in beetles fed on different plant species revealed that the accumulation rate for a given GLS can depend on the food plant. These findings indicated that the plant’s GLS composition can influence GLS sequestration in this species. However, whether rapid GLS sequestration can also prevent hydrolysis of ingested GLS in *P. striolata* is unknown. Several attempts to rear *P. striolata* in the laboratory for further studies failed, but we successfully established a laboratory colony of the horseradish flea beetle, *Phyllotreta armoraciae* (Koch) (Chrysomelidae), which we use as a model species to investigate the mechanism and function of GLS sequestration in the genus *Phyllotreta*.

Although *P. armoraciae* is monophagous on horseradish (*Armoracia rusticana*) in nature, the beetles accept several other Brassicaceae plant species as food in the laboratory (Nielsen 1978; Nielsen et al. 1979; Vig and Verdyck 2001). While beetles feed primarily on the leaf blade, larvae mine the petioles until they pupate in the soil (Vig 2004). In the laboratory, we rear *P. armoraciae* on a *Brassica juncea* cultivar that contains the same major GLS as horseradish, i.e. allyl GLS (Agneta et al. 2014; Beran et al. 2014). Preliminary studies revealed the presence of allyl GLS in all *P. armoraciae* life stages and showed that GLS were transferred from larvae through metamorphosis to the adult stage (Körnig 2015).

Here, we focused on GLS sequestration in adult *P. armoraciae* beetles. To elucidate where sequestered GLS are stored in *P. armoraciae*, we analyzed the distribution of sequestered GLS in the adult body. To understand how GLS levels and composition in food plants affect GLS sequestration, we performed a feeding experiment with newly emerged adults and three *Arabidopsis thaliana* lines. We used the *Arabidopsis* Col-0 wild type and two mutants in the Col-0 background. The plant lines differ about four-fold in total GLS levels and have different compositions of aliphatic and indolic GLS (Sønderby et al. 2007; Zhao et al. 2002). Specifically, we asked the following questions: (1) How are the GLS levels and composition in adult *P. armoraciae* beetles affected by ingested GLS? (2) Are ingested GLS selectively sequestered and metabolized? (3) Do *P. armoraciae* adults selectively excrete GLS? and (4) Does the metabolic fate of ingested GLS in *P. armoraciae* depend on GLS type, the total ingested GLS amount (influenced by the GLS level in the plant), and the GLS composition in the food plants?

## Methods and Materials

### *P. armoraciae Rearing*

The laboratory culture of *P. armoraciae* was established in 2012 using beetles collected from horseradish plants in Laasdorf, Thuringia, Germany. Adult *P. armoraciae* beetles were reared on three- to four-week old potted *Brassica juncea* cv. Bau Sin plants (Known-You Seed Co. Ltd., Kaohsiung, China) in a controlled environment chamber at 24 °C, 60% relative humidity, and a 14:10 h light:dark cycle. After one week, plants with eggs were transferred to a separate cage for larval development. Three weeks later, any remaining plant material was removed, and the soil containing pupae was kept in plastic containers (9 L volume, Lock&Lock, Seoul, South Korea) until adults emerged. Field-collected beetles were added to the colony every year to prevent an inbreeding depression. The experiments described here were carried out between 2014 and 2017.

### Localization of Sequestered GLS in *P. armoraciae* Beetles

To determine where sequestered GLS are stored in *P. armoraciae* beetles, we dissected adults collected from the laboratory colony. First, we collected hemolymph by inserting a thin glass capillary into the hemocoel between thorax and abdomen, and then separately collected head, legs, elytra, hindwings, thorax, integument, gut, fat body, and reproductive organs. Hemolymph and tissues of five males and five females were pooled in 500 μL of 80% (v/v) methanol on ice, and samples were stored at −20 °C until GLS analysis. Dissected tissues were homogenized using metal beads (2.4 mm diameter, Askubal, Korntal-Münchingen, Germany) using a TissueLyser II (QIAGEN, Hilden, Germany). After adding 50 nmol of 4-hydroxybenzyl GLS (sinalbin) as an internal standard to each sample, GLS were extracted, analyzed by HPLC-UV at 229 nm and quantified as previously described in Beran et al. (2014). The distribution of GLS in hemolymph and different beetle tissues was expressed relative to the total GLS amount detected in all samples, which was set to 100%.

### Design of the GLS Sequestration Experiment with *P. armoraciae* and *A. thaliana*

To determine how ingested GLS affect sequestration and excretion in *P. armoraciae*, we performed a feeding experiment with newly emerged beetles and three different *A. thaliana* genotypes that differ about four-fold in GLS contents and in their GLS composition. We used the Col-0 wild type of *Arabidopsis* that mainly produces 4-methylsulfinylbutyl (4MSOB) GLS and other methylsulfinylalkyl GLS as well as minor amounts of indolic GLS, and two double knock-out mutants, *myb28myb29* (*myb*) and *cyp79b2cyp79b3 (cyp)* in the Col-0 background, which are devoid of aliphatic and indolic GLS, respectively (Sønderby et al. 2007; Zhao et al. 2002). *Arabidopsis* plants were cultivated in a controlled environment chamber at 21 °C, 55% relative humidity and a 10:14 h light:dark cycle.

To compare GLS in *P. armoraciae* beetles before and after feeding on *Arabidopsis*, we randomly assigned newly emerged male and female beetles to the following four treatments, newly emerged (control before feeding), fed on wild type, fed on *myb*, and fed on *cyp*, each with ten replicates. Control beetles were immediately collected in groups of five beetles, weighed, frozen in liquid nitrogen, and stored at −20 °C for GLS analysis.

For feeding, we placed five beetles together with one detached leaf from a six- to seven-week old *Arabidopsis* plant into a Petri dish (60 mm diameter, Greiner Bio-One, Frickenhausen, Germany). The leaf petiole was inserted into a 0.2 mL reaction tube containing 0.1 mL ultrapure water to prevent wilting. Adults were provided with a new leaf taken from an undamaged *Arabidopsis* plant every day until day five, and were then starved for one additional day before sampling as described for the newly emerged adults.

To calculate how much plant tissue the beetles had ingested, we weighed each leaf before and after feeding. Because we noticed that the fresh weight of detached leaves increased by 23 ± 3% for *Arabidopsis* wild type, 22 ± 3% for *myb* mutant, and 15 ± 2% for *cyp* mutant, under our conditions (mean ± SD, *N* = 8–9), we corrected the initial leaf weight before calculating the amount of fed plant tissue. Fed leaves were frozen in liquid nitrogen and stored at −20 °C until they were freeze-dried for later GLS analysis. Feces were collected every day using 100 μL of ultrapure water per Petri dish. Each aqueous feces sample was mixed with 300 μL pure methanol (purity ≥99.9%, Carl Roth GmbH & Co. KG, Karlsruhe, Germany) and stored at −20 °C until GLS extraction.

*P. armoraciae* beetles were homogenized in 1 mL 80% (v/v) methanol containing 50 nmol sinalbin using a plastic pestle. Freeze-dried *Arabidopsis* leaves were homogenized using metal beads to a fine plant powder that was extracted with 1 mL 80% (v/v) methanol containing 50 nmol sinalbin. Feces samples were homogenized with metal beads and the volume was adjusted to 1 mL using 80% (v/v) methanol containing the internal standard sinalbin. The GLS extraction, analysis and quantification was done as described in Beran et al. (2014).

*Question 1: How are the GLS levels and composition in adult P. armoraciae beetles affected by ingested GLS?* The GLS compositions in the three *Arabidopsis* lines used in our sequestration experiment are shown in Table S1. We compared the individual and total GLS amounts (in nmol per beetle) and concentrations (in nmol per mg beetle) in newly emerged beetles with those in fed beetles by different statistical methods depending on the variance homogeneity and the normality of residuals. Comparisons by Student’s *t* test, Mann-Whitney *U* test, and analysis of variance (ANOVA) were carried out in Sigma Plot 11.0 (Systat Software, Inc., Erkrath, Germany). Analyses using the method of generalized least squares were done in R 3.5.1 (nlme package, Pinheiro et al. 2019; R Core Team 2018). If necessary, data were transformed prior to analysis. For data analyzed with the generalized least squares method, the varIdent variance structure was applied, allowing each group to have a different variance. The *P* value was obtained by removing the explanatory variable and comparing both models using a likelihood ratio test (Zuur et al. 2009). Factor level reductions were used to reveal significant differences between groups (Crawley 2013). Details of statistical methods are provided in Supplementary Tables S2 and S3.

Since the amounts of previously sequestered allyl GLS were lower in fed beetles than in newly emerged beetles, we examined the influence of the total ingested GLS amount and the food plant on the allyl GLS levels in beetles and feces, respectively, by analysis of covariance (ANCOVA) in R (R Core Team 2018). The total ingested GLS amount was calculated based on the ingested amount of leaf tissue and the corresponding GLS concentration in each fed leaf. Although GLS are unevenly distributed in *Arabidopsis* rosette leaves (Shroff et al. 2008), adult feeding damage was randomly distributed across leaves in our experiment (Fig. S1). In both analyses, the ingested GLS amount per beetle was log-transformed. Allyl GLS amounts per beetle were log-transformed, and excreted allyl GLS amounts per beetle were square-root transformed in order to achieve homogeneity and normality of the residuals. Factor level reductions were used to reveal significant differences between groups (Crawley 2013). To elucidate which proportion of the lost allyl GLS was excreted, we expressed the allyl GLS amount detected in the feces relative to the lost allyl GLS amount in adults, which was set to 100%.

*Question 2: Are ingested GLS from Arabidopsis wild type leaves selectively sequestered and metabolized in P. armoraciae adults?* To determine whether *P. armoraciae* accumulated individual GLS from *Arabidopsis* wild type leaves selectively, we expressed the concentration of each GLS in adults relative to the average concentration in feeding-damaged leaves (set to 1). The relative (fold) accumulation of different GLS in *P. armoraciae* was compared using the generalized least squares method (nlme package, Pinheiro et al. 2019). Data were square-root-transformed prior to analysis.

The analysis of the relative accumulation of individual GLS from *Arabidopsis* wild type leaves in *P. armoraciae* revealed a disproportionately high accumulation of 4-methylthiobutyl (4MTB) GLS in beetles. In addition, these beetles contained significantly higher amounts of 3-butenyl (3But) GLS, although this GLS was not present in their food plant. To determine whether *P. armoraciae* converts 4MSOB GLS, the major aliphatic GLS in *Arabidopsis* wild type leaves, to 4MTB GLS and 3But GLS, we fed newly emerged adults with an aqueous solution containing 10 nmol 4MSOB GLS (purchased from Phytoplan, Heidelberg, Germany). We placed newly emerged beetles in a Petri dish with a 0.2 μl droplet containing the GLS, or pure water as a control, and observed each beetle until it had finished drinking the droplet under a microscope. To allow adults to metabolize the ingested 4MSOB GLS, they were fed for three days with detached *B. juncea* leaves, which do not contain 4MSOB GLS and 4MTB GLS (Beran et al. 2014). Afterwards, adults were frozen in liquid nitrogen and stored at −20 °C until GLS extraction. We analyzed four and six replicates for the control and 4MSOB GLS treatments, respectively, each consisting of five adults. Because the 4MSOB GLS solution fed to beetles contained a small amount of 4MTB GLS as contaminant, we compared the ingested 4MTB GLS amount (present in the fed 4MSOB GLS solution) with the amount detected in fed beetles by Student’s *t* test. Because 3But GLS was detected in control and fed beetles, we compared the levels in both groups by Student’s *t* test. In addition to 4MSOB GLS, *Arabidopsis* contains also other methylsulfinylalkyl GLS, including 7-methylsulfinylheptyl GLS and 8-methylsulfinyloctyl GLS, which might be converted to the corresponding methylthioalkyl GLS. Since the chromatographic conditions used for the GLS analysis in beetles and feces did not allow the detection of 7-methylthioheptyl- and 8-methylthiooctyl GLS, we analyzed several samples by HPLC-UV at 229 nm using a modified solvent gradient of 0.2% formic acid (solvent A) and acetonitrile (solvent B): 1.5% (v/v) B (1 min), 1.5–5% (v/v) B (5 min), 5–7% (v/v) B (2 min), 7–21% (v/v) B (10 min), 21–29% (v/v) B (5 min), 29–43% (v/v) B (7 min), 43–100% (v/v) B (0.5 min), 100% (v/v) B (2.5 min), 100 to 1.5% (v/v) B (0.1 min), and 1.5% (v/v) B (4.9 min). In addition, we determined the presence of methylthiolalkyl GLS in beetle and feces samples by liquid chromatography coupled with mass spectrometry. Chromatographic analyses (as described above) were carried out on 1100 series equipment (Agilent Technologies, Waldbronn, Germany) coupled to an Esquire 6000 ESI-Ion Trap mass spectrometer (Bruker Daltonics, Bremen, Germany) operated in positive ionization mode in the range of *m/z* 60–1000, with a skimmer voltage of 52.8 V, capillary exit voltage of 117.3 V, capillary voltage of 3000 V, nebulizer pressure of 35 psi, drying gas of 11 L/min, and gas temperature of 330 °C. Elution was accomplished at a flow rate of 1 mL/min at 25 °C under chromatographic conditions as described above. Flow coming from the column was diverted at a ratio of 4:1 before reaching the electrospray ionization (ESI) unit. We detected 3-methylthiopropyl-, 7-methylthioheptyl- and 8-methylthiooctyl GLS as desulfo-GLS in samples by comparing the retention times, UV spectra, mass spectra and in-source fragmentation patterns to those of isolated standards (Brown et al. 2003). The presence of 5-methylthiopentyl GLS was analyzed according to its UV spectrum, mass spectrum, and in-source fragmentation pattern.

*Question 3: Do P. armoraciae adults excrete GLS selectively?* The amounts of individual and total GLS detected in feces were compared by different statistical methods as described above. Details of statistical analyses are provided in Table S4. To determine whether beetles excreted previously sequestered allyl GLS and ingested GLS selectively, we compared the amounts of allyl GLS, 4MSOB GLS, and indol-3-ylmethyl (I3M) GLS excreted by adults fed on *Arabidopsis* wild type leaves using a linear mixed effects model. The lme function (Pinheiro et al. 2019) was applied to account for the different beetle groups. GLS and day were treated as fixed effects, and beetle groups as random effect. The GLS amount was log-transformed prior to analysis. *P* values and significant differences between groups were obtained as described above.

*Question 4: Does the metabolic fate of ingested GLS in P. armoraciae depend on GLS type, total ingested GLS amount, and the GLS composition in the food plant?* To analyze the metabolic fate of ingested aliphatic and indolic GLS, we calculated the percentage of sequestered and excreted aliphatic and indolic GLS relative to the total amount of ingested aliphatic and indolic GLS, respectively (set to 100%). Because a low background of indolic GLS was present in newly emerged adults, we subtracted the average amount of each indolic GLS detected in newly emerged beetles from the corresponding GLS amounts detected after feeding on *Arabidopsis*. To analyze whether the total ingested GLS amount (covariable), the GLS type (aliphatic or indolic GLS as explanatory variable) or the GLS composition (*Arabidopsis* line as explanatory variable) affect the metabolic fate of ingested GLS, we performed ANCOVA or analyzed the data using the method of generalized least squares, or linear mixed effects models (nlme package, Pinheiro et al. 2019) with GLS ingestion and GLS type as fixed effects and beetle groups feeding on a certain plant as random intercept. ANCOVA analyses were conducted with type II variance partitioning of the car library (Fox and Weisberg 2011) to adjust each effect for other effects (Kabacoff 2011). If necessary, data were transformed prior to analysis. To determine the appropriate variance structure for the generalized least squares analyses, models fitted with different variance structures were compared based on the Akaike information criterion (AIC) (Zuur et al. 2009). *P* values were obtained as described above. We used the total ingested GLS amount instead of the ingested amounts of aliphatic and indolic GLS in our analyses, respectively, because a Spearman’s rank correlation coefficient analysis showed a strong positive correlation between the total ingested GLS amount, the ingested aliphatic GLS amount and the ingested indolic GLS amount, respectively (rho ≥ 0.770, *P* ≤ 0.014; Fig. S2). Details of statistical analyses are given in Table 2.

## Results

*Localization of sequestered GLS* After hemolymph collection and dissection of *P. armoraciae* beetles, we found different quantities of GLS in all tissues. The highest proportion of GLS was detected in the hemolymph, which contained about one third of the total detected GLS. High proportions of GLS were also found in the elytra (22%), the legs (17%), and the head (10%), whereas only traces of GLS were found in reproductive organs and the fat body (Table 1).

**Table 1:** Distribution of sequestered glucosinolates (GLS) in *P. armoraciae* adults

Body part/Tissue	Percentage of the total detected GLS
Hemolymph	29
Elytra	22
Legs	17
Head	10
Thorax	9
Hindwings	4
Gut	4
Integument	3
Reproductive organs	1
Fat body	1

Results are based on one sample derived from 5 females and 5 males

**Fig. 1:**
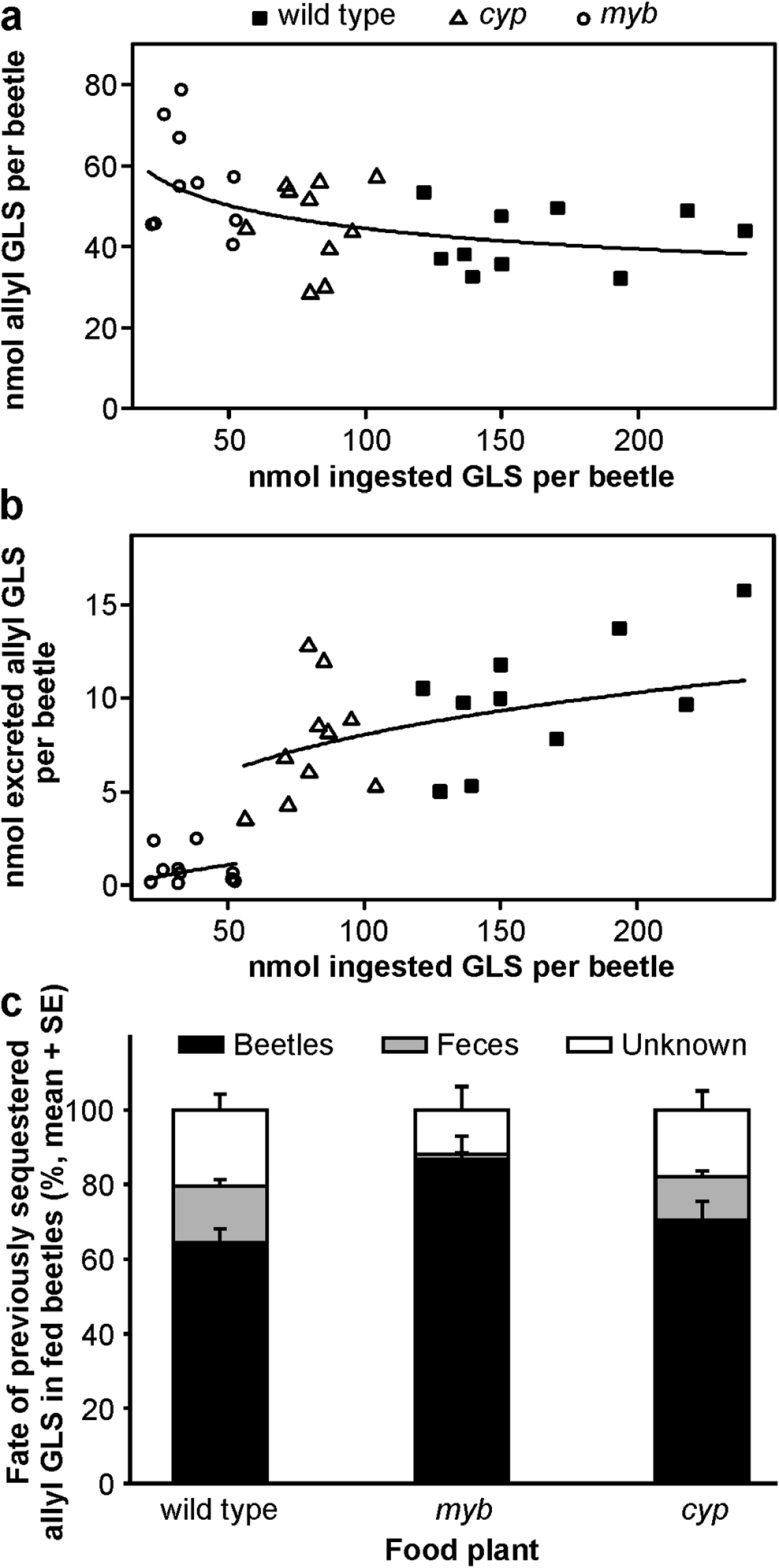
Fate of previously sequestered allyl glucosinolate (GLS) in *P. armoraciae* beetles fed on different *Arabidopsis* lines for five days and starved for one day. **a** Relationship between the total ingested GLS amount and the amount of previously sequestered allyl GLS per beetle. **b** Relationship between the total ingested GLS amount and the amount of excreted allyl GLS per beetle. The statistical analyses were performed by ANCOVA using the total ingested GLS amount as a covariable. In the first analysis (**a**), both the total ingested GLS amount and allyl GLS amount were log transformed prior to analysis. In the second analysis (**b**), the total ingested GLS amount and the excreted allyl GLS amount was log transformed and square-root transformed, respectively, prior to analysis. The final estimates were back-transformed to their original scale, and plotted with regression lines. Data series that are not significantly different from each other were combined for regression line plotting. **c** Percentage of allyl GLS in fed beetles and feces relative to the allyl GLS amount in newly emerged beetles (*N* = 10). *myb*, *Arabidopsis myb28myb29* double knockout mutant; *cyp*, *Arabidopsis cyp79b2cyp79b3* double knockout mutant

*Question 1: How are the GLS levels and composition in adult P. armoraciae beetles affected by ingested GLS?* The concentrations and amounts of GLS detected in newly emerged and fed beetles are summarized in Tables S2 and S3, respectively. Newly emerged *P. armoraciae* adults contained mainly allyl GLS and minor amounts of 3But GLS and indolic GLS. This GLS composition largely corresponds to that in the rearing plant *B. juncea* (Beran et al. 2014). After feeding on different *Arabidopsis* lines, the total GLS concentrations and amounts in fed adults did not differ from those in newly emerged adults (GLS concentration: generalized least squares method, likelihood ratio = 6.309, *P* = 0.098; GLS amount: ANOVA, *F* = 1.108, *P* = 0.359). However, the GLS compositions in fed adults differed because of an accumulation of aliphatic and/or indolic GLS. This GLS uptake was balanced with a decrease of previously sequestered allyl GLS in beetles (Table S3). The allyl GLS levels in beetles were negatively correlated with the amount of ingested GLS, and did not depend on the food plant (Fig. 1a; ANCOVA, ingested GLS amount: *F* = 8.391, *P* = 0.007; plant: *F* = 0.456, *P* = 0.639; ingested GLS amount × plant: *F* = 0.136, *P* = 0.873). To determine whether beetles regulate their endogenous GLS levels by excreting GLS, we quantified the amounts of allyl GLS in feces (Table S4). Allyl GLS excretion was positively correlated with the ingested GLS amount and, in addition, depended on the food plant, because *myb*-fed adults excreted significantly less allyl GLS than wild type- or *cyp*-fed adults (Fig. 1b; ANCOVA, ingested GLS amount: *F* = 97.303, *P* < 0.001, plant: *F* = 7.852, *P* = 0.002; ingested GLS amount × plant: *F* = 2.527, *P* = 0.101). In total, we recovered up to 40% of the lost allyl GLS (set to 100%) in feces (Fig. 1c).

**Fig. 2:**
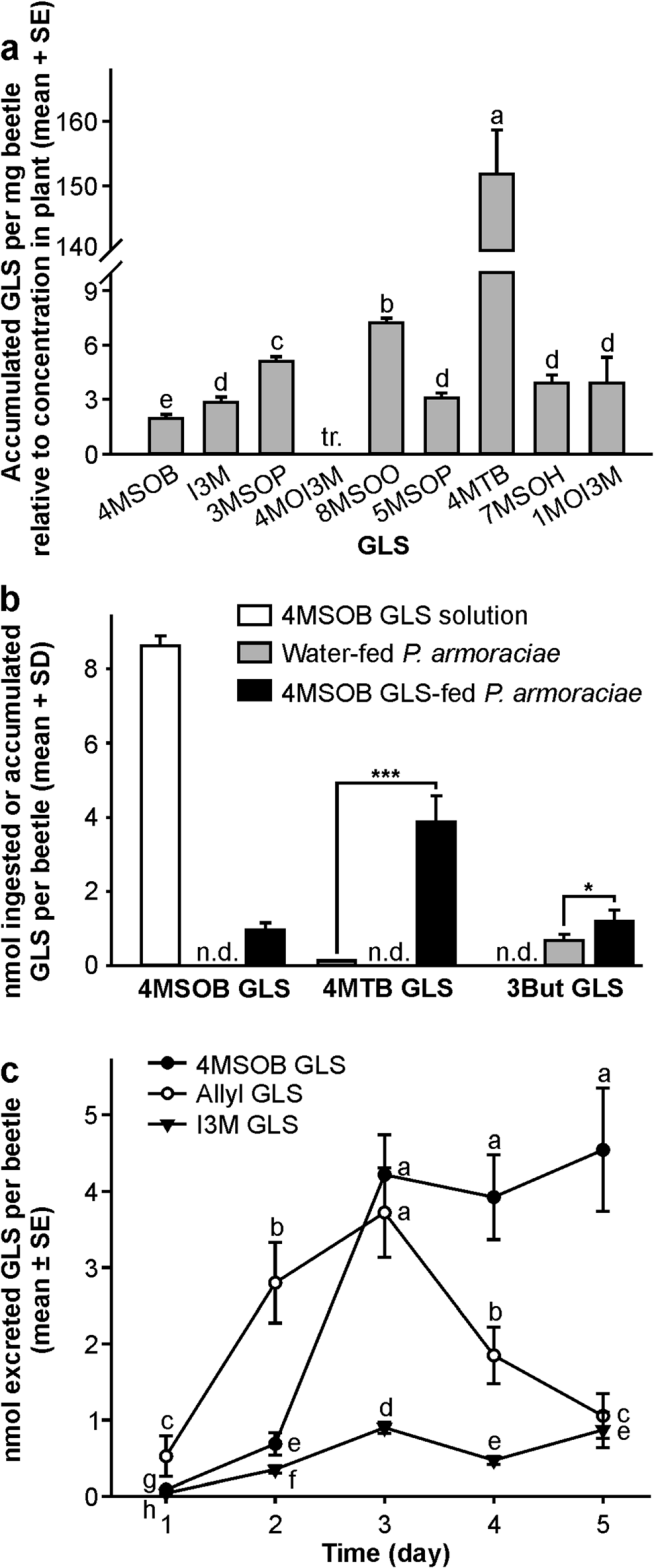
Accumulation, metabolism and excretion of ingested GLS in *P. armoraciae* adults. **a** GLS accumulation pattern in *P. armoraciae* adults after five days feeding on *Arabidopsis* wild type leaves and one day starvation. To compare the accumulation of different GLS in *P. armoraciae*, the concentration of each GLS in adults is expressed relative to that in leaves (set to 1). GLS are sorted from the highest to the lowest concentration in *Arabidopsis*. Bars labeled with different letters are significantly different (generalized least squares method, *P* < 0.05, *N* = 10). **b** Metabolism of sequestered 4MSOB GLS in *P. armoraciae* adults. Adults were fed with an aqueous 4MSOB GLS solution or water as a control, and harvested for GLS extraction after feeding on *B. juncea* for three days (*N* = 4–6). The amounts of 4MTB GLS and 3But GLS between different groups were compared by Student’s *t* test. A small amount of 4MTB GLS was detected as a contaminant in the 4MSOB GLS solution, but 4MTB GLS amounts in 4MSOB GLS-fed adults were significantly higher (*t* = −8.241, *P* ≤ 0.001). **c** Time course of GLS excretion over five days feeding on *Arabidopsis* wild type leaves. The graph shows the excreted amounts of previously sequestered allyl GLS and newly ingested 4MSOB GLS and I3M GLS on each day. Different letters indicate significant differences between different days and GLS (linear mixed effects model, *P* < 0.05, *N* = 10). 3But, 3-butenyl; 4MSOB, 4-methylsulfinylbutyl; 4MTB, 4-methylthiobutyl; 8MSOO, 8-methylsulfinyloctyl; 3MSOP, 3-methylsulfinylpropyl; 5MSOP, 5-methylsulfinylpentyl; 7MSOH, 7-methylsulfinylheptyl; I3M, indol-3-ylmethyl; 4MOI3M, 4-methoxyindol-3-ylmethyl; 1MOI3M, 1-methoxyindol-3-ylmethyl; tr., traces; n.d., not detected; *P < 0.05; ***P < 0.001

*Question 2: Are ingested GLS from Arabidopsis wild type leaves selectively sequestered and metabolized in P. armoraciae adults? P. armoraciae* accumulated almost all GLS present in *Arabidopsis*, but at widely divergent efficiencies (Fig. 2a; generalized least squares method, likelihood ratio = 127.463, *P* < 0.001). While most GLS were concentrated between two and seven-fold in adults, the 4MTB GLS concentration was 152-fold higher in adults than in leaves. Since these 4MTB GLS amounts in beetles cannot be explained by direct accumulation from the food plant (ingestion of 321 mg wild type leaf per beetle would be necessary, but at most 70 mg of plant tissue was ingested per beetle), we hypothesized that *P. armoraciae* can convert sequestered 4MSOB GLS, the major aliphatic GLS in *Arabidopsis* wild type leaves, to 4MTB GLS. To test this hypothesis, we fed *P. armoraciae* with an aqueous 4MSOB GLS solution or water as a control. Three days later, we detected about four times more 4MTB GLS than 4MSOB GLS in these adults, whereas both GLS were below the detection limit in the control adults (Fig. 2b). Although a minor amount of 4MTB GLS was present in the 4MSOB GLS solution as a contaminant, significantly larger amounts of 4MTB GLS were detected in 4MSOB GLS-fed beetles (Student’s *t* test, *t* = −8.241, *P* ≤ 0.001). In addition, we found significantly more 3But GLS in 4MSOB GLS-fed adults than in the corresponding control adults, which indicates that *P. armoraciae* metabolize 4MSOB GLS to minor amounts of 3But GLS (Fig. 2b; Student’s *t* test, *t* = −3.044, *P* = 0.016).

Since *Arabidopsis* contains other methylsulfinylalkyl GLS (Table S1), we searched for the corresponding methylthioalkyl GLS in beetles and feces, but detected only traces of 3-methylthiopropyl GLS in beetles.

*Question 3: Do P. armoraciae adults excrete GLS selectively?* A comparison of the GLS profiles in beetles and feces revealed that two GLS present in beetles, i.e. 3But- and 4MTB GLS, were not excreted (Tables S3, S4). We then analyzed the time-course of GLS excretion in wild type-fed adults by comparing the excreted amounts of allyl GLS with those of ingested 4MSOB GLS and I3M GLS on each day (Fig. 2c). We observed divergent excretion patterns for all three GLS, and found that the excreted amounts of each GLS depended on the day and the GLS type (linear mixed effects model, day: likelihood ratio = 97.331, *P* < 0.001; GLS: likelihood ratio = 80.494, *P* < 0.001; day × GLS: likelihood ratio = 44.642, *P* < 0.001). During the first two days of feeding, adults excreted primarily allyl GLS. The amounts of excreted 4MSOB GLS and I3M GLS increased over time and during the last two days, adults excreted significantly more 4MSOB GLS than allyl GLS.

**Fig. 3:**
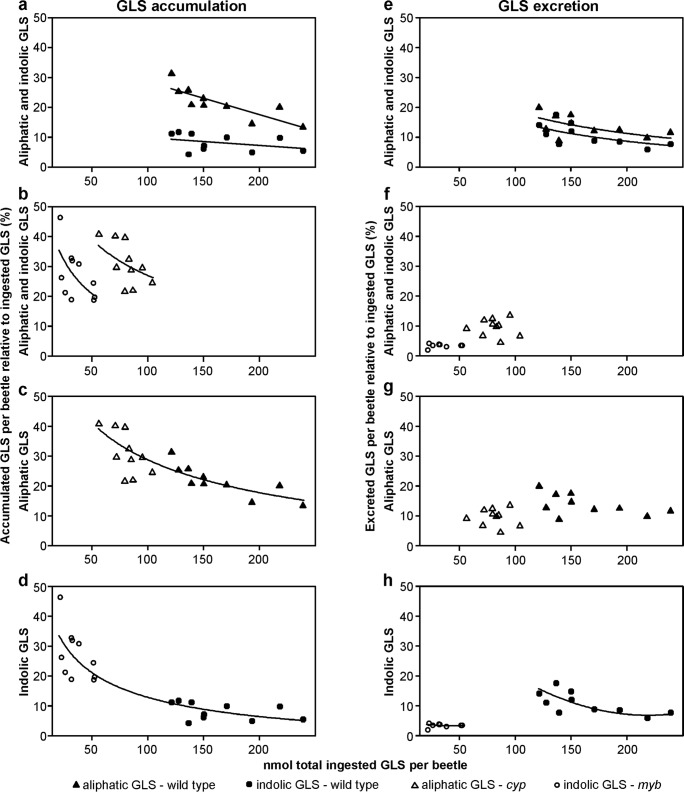
Metabolic fate of the ingested aliphatic and indolic GLS in *P. armoraciae* adults. The amount of aliphatic and/or indolic GLS ingested from *Arabidopsis* wild type, *myb*, and *cyp* rosette leaves, respectively, was set to 100%, and the corresponding percentages of aliphatic and indolic GLS detected in beetles and feces were calculated. The plots show the accumulated and excreted proportions of ingested aliphatic and/or indolic GLS by *P. armoraciae* adults relative to the total ingested GLS amount per beetle. **a** Accumulation of aliphatic and indolic GLS ingested from *Arabidopsis* wild type. **b** Accumulation of aliphatic and indolic GLS ingested from the *cyp* or *myb* mutant, respectively. **c** Accumulation of aliphatic GLS ingested from wild type plant and *cyp* mutant, respectively. **d** Accumulation of indolic GLS ingested from wild type plant and *myb* mutant, respectively. **e** Excretion of aliphatic and indolic GLS ingested from *Arabidopsis* wild type. **f** Excretion of aliphatic and indolic GLS ingested from *cyp* and *myb* mutant, respectively. **g** Excretion of aliphatic GLS ingested from wild type plant and *cyp* mutant, respectively. **h** Excretion of indolic GLS ingested from wild type plant and *myb* mutant, respectively. The statistical analyses were performed by ANCOVA, generalized least squares method, or linear mixed effects models using the total ingested GLS amount per beetle as covariable, with linear or quadratic regression. If necessary, data were transformed prior to analysis. The final estimates were back-transformed to their original scale, and plotted with regression lines. Data series that are not significantly different from each other were combined for regression line plotting. For methods and results of the statistical analyses, refer to Table 2. Aliphatic GLS: sum of 3MSOP GLS, 4MSOB GLS, 4MTB GLS, 5MSOP GLS, 7MSOH GLS, 8MSOO GLS; indolic GLS: sum of I3M GLS, 1MOI3M GLS, 4MOI3M GLS. For plant and GLS abbreviations, refer to legend of Figs. 1 and 2, respectively

*Question 4: Does the metabolic fate of ingested GLS in P. armoraciae depend on GLS type, total ingested GLS amount, and GLS composition in the food plant?* To analyze the metabolic fate of ingested GLS, we quantified how much of the total ingested aliphatic and indolic GLS were accumulated and excreted, respectively. In general, we found that adults accumulated and excreted significantly higher percentages of ingested aliphatic than indolic GLS (Fig. 3a, b, e, f, Table 2). However, *P. armoraciae* accumulated higher proportions of ingested GLS from mutants than from wild type plants (Fig. 3c, d, Table 2). This higher accumulation of ingested GLS from mutant leaves was due to the lower total ingested GLS amount, and not due to the different GLS compositions in mutant and wild type leaves (Fig. 3c, d, Table 2). In addition, the total ingested GLS amount negatively affected the accumulation rate of GLS in beetles (Fig. 3a–c, Table 2), as well as the excreted GLS proportion in wild type-fed adults (Fig. 3e–g, Table 2). However, the GLS composition, i.e. the presence of both GLS types, or only one GLS type in the food plant, affected the proportion of excreted GLS, but not the proportion of accumulated GLS (Fig. 3c, d, g, h; Table 2). Specifically, adults excreted significantly higher proportions of ingested GLS from wild type than from mutant leaves (Fig. 3g, h).

**Table 2:** Methods and results of statistical analyses of metabolic fate of ingested aliphatic and indolic GLS in *P. armoraciae* adults

Metabolic fate	Comparison	Statistical method	Equation form	Variance structure	Variable	Transformation	Statistics	*P* value
Accumulation	Aliphatic and indolic GLS from *Arabidopsis* wild type (Fig. 3a)	Linear mixed effects model	Linear	–	Accumulated percentage	–	–	–
					Ingested GLS amount	–	*LR* = 8.790	= 0.003
					GLS type	–	*LR* = 27.778	< 0.001
					Interaction	–	*LR* = 6.860	= 0.009
	Aliphatic GLS from *cyp* mutant, indolic GLS from *myb* mutant (Fig. 3b)	ANCOVA	Linear	–	Accumulated percentage	Arcsin-square-root	–	–
					Ingested GLS amount	Log	*F* = 7.631	= 0.013
					Plant	–	*F* = 9.160	= 0.008
					Interaction	–	*F* = 0.687	= 0.420
	Aliphatic GLS from *Arabidopsis* wild type and *cyp* mutant (Fig. 3c)	Generalized least squares	Linear	varComb (varIdent (form = ~ 1 | plant),varFixed(~ Ingested GLS amount))	Accumulated percentage	Arcsin-square-root	–	–
					Ingested GLS amount	Log	*LR* = 24.058	< 0.001
					Plant	–	*LR* = 3.625	= 0.057
					Interaction	–	*LR* = 0.150	= 0.698
	Indolic GLS from *Arabidopsis* wild type and *myb* mutant (Fig. 3d)	Generalized least squares	Linear	varPower (form = ~ log (Ingested GLS amount))	Accumulated percentage	Arcsin-square-root	–	–
					Ingested GLS amount	Log	*LR* = 26.602	< 0.001
					Plant	–	*LR* = 1.095	= 0.295
					Interaction	–	*LR* = 0.560	= 0.454
Excretion	Aliphatic and indolic GLS from *Arabidopsis* wild type (Fig. 3e)	Linear mixed effects model	Linear	–	Excreted percentage	Arcsin-square-root	–	–
					Ingested GLS amount	Log	*LR* = 5.279	= 0.022
					GLS type	–	*LR* = 13.807	< 0.001
					Interaction	–	*LR* = 3.313	= 0.069
	Aliphatic GLS from *cyp* mutant, indolic GLS from *myb* mutant (Fig. 3f)	Generalized least squares	Linear	varExp (form = ~ Ingested GLS amount | plant)	Excreted percentage	–	–	–
					Ingested GLS amount	–	*LR* = 0.002	= 0.964
					Plant	–	*LR* = 15.311	< 0.001
					Interaction	–	*LR* = 0.321	= 0.571
	Aliphatic GLS from *Arabidopsis* wild type and *cyp* mutant (Fig. 3g)	ANCOVA	Linear	–	Excreted percentage	–	–	–
					Ingested GLS amount	–	*F* = 3.413	= 0.082
					Plant	–	*F* = 8.194	= 0.010
					Interaction	–	*F* = 0.142	= 0.711
	Indolic GLS from *Arabidopsis* wild type and *myb* mutant (Fig. 3h)	Generalized least squares	Quadratic	varExp (form = ~ Ingested GLS amount | plant)	Excreted percentage	–	–	–
					Ingested GLS amount	–	*LR* = 6.809	0.009
					Plant	–	*LR* = 8.359	0.004
					Ingested GLS amount: Plant Interaction	–	*LR* = 6.095	0.014
					Ingested GLS amount^2^	–	*LR =* 5.659	0.017
					Ingested GLS amount^2^:Plant Interaction	–	*LR =* 0.062	0.803

*LR*, likelihood ratio

In total, we recovered 35% of the ingested aliphatic GLS and 19% of the indolic GLS ingested from *Arabidopsis* wild type leaves, 40% of the aliphatic GLS ingested from *cyp* leaves, and 31% of the indolic GLS ingested from *myb* leaves (Table 3). The metabolic fate of the remaining ingested GLS is unknown.

**Table 3:** Metabolic fate of the ingested aliphatic and indolic GLS in *P. armoraciae* adults that fed on leaves of different *Arabidopsis* lines for 5 days

	Mean percentage^1^ ± SD; *N* = 10			
Metabolic fate	Aliphatic GLS wild type	Indolic GLS wild type	Aliphatic GLS *cyp*	Indolic GLS *myb*
Accumulation	21.5 ± 5.3	8.2 ± 2.9	30.9 ± 7.3	27.1 ± 8.7
Excretion	13.6 ± 3.6	10.8 ± 3.8	9.5 ± 2.9	3.4 ± 0.6
Total recovery^2^	35.1 ± 8.1	19.0 ± 4.0	40.3 ± 7.9	30.5 ± 8.3

^1^The amount of aliphatic and/or indolic GLS ingested from *Arabidopsis* wild type, *myb*, and *cyp* mutant, respectively, was set to 100%. The corresponding percentages of aliphatic and indolic GLS in adults (accumulation) and feces (excretion) were calculated. ^2^The total recovery corresponds to the recovered proportion of ingested GLS, which was detected in adults (accumulation) and feces (excretion). Aliphatic GLS: sum of 3MSOP GLS, 4MSOB GLS, 4MTB GLS, 5MSOP GLS, 7MSOH GLS, 8MSOO GLS; indolic GLS: sum of I3M GLS, 1MOI3M GLS, 4MOI3M GLS. For plant and GLS abbreviations, refer to legend of Figs. 1 and 2, respectively

## Discussion

In this study, we analyzed the metabolic fate of both ingested and previously sequestered GLS in adult *P. armoraciae*. Our first major finding was that GLS sequestration in *P. armoraciae* saturates at levels of about 35 nmol GLS per mg beetle. To balance the accumulation of new aliphatic and indolic GLS, previously sequestered allyl GLS is lost (Table S3). This finding differs from previous results with *P. striolata* that showed that adults lost about 30% of their total sequestered GLS after feeding on *myb* plants for 18 days, whereas the total GLS levels increased three-fold when adults were shifted from *Arabidopsis myb* plants to *B. juncea* as food source (Beran et al. 2014). These findings suggest differences in the regulation of the total GLS levels in *P. armoraciae* and *P. striolata*, a topic that could be explored in comparative feeding studies with both *Phyllotreta* species.

One mechanism used by *P. armoraciae* to regulate its endogenous GLS levels is the excretion of sequestered GLS. A similar regulatory mechanism was observed in cabbage aphids, where decreasing GLS levels in juveniles developing into winged adult aphids were associated with the excretion of GLS (Kazana et al. 2007). The excretion of intact GLS implies that there is no plant myrosinase activity in the feces. This finding is remarkable given that ingested plant myrosinase enzyme was found to be highly resistant against digestive proteolysis in larvae of the generalist African cotton leafworm, *Spodoptera littoralis* (Boisduval) (Noctuidae) (Vassão et al. 2018). It will be interesting to elucidate whether plant defense proteins are digested more efficiently in the specialist *P. armoraciae* beetles than in the generalist *S. littoralis* larvae, or whether sequestering beetles inhibit plant myrosinase activity by other mechanisms.

*P. armoraciae* accumulated almost all types of GLS present in *Arabidopsis* wild type leaves, but some GLS were accumulated more than others. For example, *P. armoraciae* sequestered significantly more 8-methylsulfinyloctyl GLS than 7-methylsulfinylheptyl GLS (Fig. 2a). The different GLS accumulation efficiencies could be the result of selective GLS uptake from the gut and/or different rates of GLS metabolism and excretion. Similar to our previous study with *P. striolata*, we detected larger amounts of 4MTB GLS than could be accounted for by dietary intake in *P. armoraciae* (Beran et al. 2014). Here we show that adults metabolize 4MSOB GLS to 4MTB GLS and, in addition, to minor amounts of 3But GLS (Fig. 2b). In contrast, we found no evidence for a conversion of long-chain methylsulfinylalkyl GLS in *P. armoraciae*. This metabolism of 4MSOB GLS to 4MTB GLS and 3But GLS does not seem to be common for GLS-sequestering species since we did not observe the formation of 4MTB GLS and 3But GLS in the cabbage stem flea beetle *Psylliodes chrysocephala* (L.) (Chrysomelidae) (Fig. S3). A chemical reduction of methylsulfinylalkyl GLS, isothiocyanates, and nitriles to the corresponding methylthioalkyl metabolites was previously observed in several bacterial strains, but the functional significance of these metabolic conversions in bacteria and *P. armoraciae* remains unknown (Luang-In et al. 2014; Narbad and Rossiter 2018).

In addition to selective GLS accumulation and metabolism, we also observed selective excretion of GLS in *P. armoraciae*. Although 4MTB GLS is more abundant in adults than 4MSOB GLS, no 4MTB GLS was detected in feces, suggesting metabolic conversion of the ingested GLS. However, we cannot exclude that 4MTB GLS was further metabolized or decomposed after excretion and was therefore not detected. Furthermore, after feeding on wild type *Arabidopsis* for several days, adults selectively excreted the more recently ingested 4MSOB GLS rather than the previously stored allyl GLS (Fig. 2c). The excretion of previously sequestered GLS is likely mediated by the Malpighian tubules, the major organ responsible for the excretion of xenobiotics and plant toxins from the insect hemolymph (Dermauw and Van Leeuwen 2014; Maddrell and Gardiner 1976; Ruiz-Sanchez and O'Donnell 2015). GLS excretion may either be an active process as demonstrated for nicotine in the tobacco hornworm, *Manduca sexta* (L.) (Sphingidae), or may occur passively as observed for the cardiac glycoside ouabain in two polyphagous orthopterans (migratory locust *Locusta migratoria* (L.) (Acrididae) and variegated grasshopper *Zonocerus variegatus* (L.) (Pyrgomorphidae)) and the ouabain-sequestering milkweed bug, *Oncopeltus fasciatus* (Dallas) (Lygaeidae) (Gaertner et al. 1998; Meredith et al. 1984; Rafaeli-Bernstein and Mordue 1979; Rafaeli-Bernstein and Mordue 1978). Whether ingested GLS detected in the feces had previously been taken up into the body or had simply passed through the digestive system cannot be determined from the current study.

The metabolic fate of ingested GLS in *P. armoraciae* was influenced by several factors, i.e. GLS type, the total amount of ingested GLS (GLS level in the food plant), and GLS composition in the food plant (Fig. 3). In general, we recovered significantly less ingested indolic than aliphatic GLS, which indicates that indolic GLS were metabolized at a higher rate than aliphatic GLS in beetles or are sequestered in some unrecoverable form. GLS accumulation and excretion together accounted for the metabolic fate of up to 41% and 31% of the total ingested aliphatic and indolic GLS from *Arabidopsis*, respectively. Independent of the GLS type, the total ingested GLS amount negatively influenced the GLS recovery. In other words, when *P. armoraciae* ingested higher levels of GLS, the proportion of accumulated and excreted GLS was lower (Fig. 3). One possible explanation for this result could be a limited capacity to stabilize ingested GLS at higher concentrations. The GLS composition in the food plant represents the third factor that influenced the metabolic fate of GLS in *P. armoraciae* because beetles excreted a higher proportion of ingested GLS from *Arabidopsis* wild type than from *myb* and *cyp* mutants, respectively. Our findings suggest that the concomitant ingestion of aliphatic and indolic GLS promotes the excretion of both GLS types by an unknown mechanism. However, the metabolic fate of more than 50% of the total ingested GLS remained unexplained in our study. To elucidate the reasons for this low recovery, in future work we will test the following hypotheses: i) the plant myrosinase hydrolyzes most ingested GLS during feeding and digestion, and ii) the beetle myrosinase hydrolyses sequestered GLS in *P. armoraciae*. Alternatively, GLS may also be metabolized by other pathways. There is still much to be learned about the fate of GLS in this sequestering insect.

In conclusion, we show that GLS variability in Brassicaceae influences the composition but not the level of sequestered GLS in *P. armoraciae* beetles. Our study revealed that *P. armoraciae* developed mechanisms to maintain stable GLS levels in their bodies by balancing uptake and excretion. The ecological consequences of different GLS accumulation patterns in beetles, in particular the effects on natural enemies or on intraspecific communication, remain to be determined in future studies.

## Electronic supplementary material

ESM 1 (DOCX 284 kb)
