# Supplementary material for: Glucosinolate Abundance and Composition in Brassicaceae Influence Sequestration in a Specialist Flea Beetle
Source: J Chem Ecol. 2020 Jan 17;46(2):186–97. doi: 10.1007/s10886-020-01144-y (PMC7056735; doi:10.1007/s10886-020-01144-y)
Supplement: Supplementary file 1 — (DOCX 284 kb) [file 10886_2020_1144_MOESM1_ESM.docx]

**Supplementary material**

**for**

**Selective accumulation, metabolism, and excretion of glucosinolates in a specialist flea beetle**

Zhi-Ling Yang^A†^, Grit Kunert^B†^, Theresa Sporer^A^, Johannes Körnig^A^, Franziska Beran^A*^

*Max Planck Institute for Chemical Ecology, Hans-Knoell-Strasse 8, D-07745 Jena, Germany,*

*^A^Research Group Sequestration and Detoxification in Insects, ^B^Department of Biochemistry*

^†^These authors contributed equally to this study.

^*^Corresponding author, email: fberan@ice.mpg.de, phone: +493641571553, fax:+493641571502

**Supplementary Tables and Figures**

**Supplementary Tables**

**Table S1** Glucosinolate (GLS) concentrations of 6-week-old feeding-damaged leaves of different *Arabidopsis* lines

| **GLS side chain** | **nmol GLS per mg plant fresh weight (mean ± SD; *N* = 50)** | | |
| --- | --- | --- | --- |
|  | **wild type** | ***myb*** | ***cyp*** |
| **3MSOP** | 0.222 ± 0.069 | — | 0.173 ± 0.067 |
| **4MSOB** | 1.829 ± 0.584 | — | 1.471 ± 0.598 |
| **4MTB** | 0.049 ± 0.034 | — | 0.038 ± 0.022 |
| **5MSOP** | 0.060 ± 0.017 | — | 0.057 ± 0.016 |
| **7MSOH** | 0.019 ± 0.007 | — | 0.022 ± 0.009 |
| **8MSOO** | 0.095 ± 0.049 | — | 0.093 ± 0.056 |
| **I3M** | 0.399 ± 0.112 | 0.520 ± 0.192 | — |
| **4MOI3M** | 0.108 ± 0.036 | 0.130 ± 0.040 | — |
| **1MOI3M** | 0.002 ± 0.001 | 0.004 ± 0.002 | — |
| **Total** | 2.781 ± 0.795 | 0.653 ± 0.215 | 1.855 ± 0.725 |

—, not detected. wild type, *Arabidopsis* Col-0; *myb*, *myb28myb29* mutant; *cyp*, *cyp79b2cyp79b3* mutant; GLS side chain abbreviations: 3MSOP, 3-methylsulfinylpropyl; 4MSOB, 4-methylsulfinylbutyl; 4MTB, 4-methylthiobutyl; 5MSOP, 5-methylsulfinylpentyl; 7MSOH, 7-methylsulfinylheptyl; 8MSOO, 8-methylsulfinyloctyl; I3M, indol-3-ylmethyl; 4MOI3M, 4-methoxyindol-3-ylmethyl; 1MOI3M, 1-methoxyindol-3-ylmethyl.

**Table S2** GLS concentrations in newly emerged *P. armoraciae* adults and after five days of feeding on three different *Arabidopsis* lines

| **GLS side chain** | **nmol sequestered GLS per mg beetle (mean ± SD (median), *N* = 10)** | | | | **Statistical method** | **Statistics** | ***P* value** |
| --- | --- | --- | --- | --- | --- | --- | --- |
|  | **Newly emerged beetles** | **Beetles fed**  **on wild type** | **Beetles fed**  **on *myb*** | **Beetles fed**  **on *cyp*** |  |  |  |
| **Allyl** | 34.107 ± 6.430  (34.339) a | 19.810 ± 1.735  (19.299) c | 26.330 ± 5.096  (25.985) b | 21.792 ± 5.003  (23.407) c | Generalized least squares | *LR* = 29.190 | < 0.001 |
| **3But** | 0.496 ± 0.285  (0.439) a | 0.760 ± 0.286  (0.780) a | 0.097 ± 0.090  (0.059) b | 0.627 ± 0.229  (0.535) a | ANOVA^1^ | *F* = 25.489 | < 0.001 |
| **3MSOP** | — | 1.133 ± 0.172  (1.206) a | — | 0.859 ± 0.146  (0.851) b | Student's *t*-test | *t* = 3.847 | = 0.001 |
| **4MSOB** | — | 3.579 ± 1.209  (3.412) | — | 3.051 ± 0.983  (3.293) | Student's *t*-test | *t* = 1.071 | = 0.298 |
| **4MTB** | — | 7.436 ± 1.067  (7.668) | — | 6.557 ± 1.108  (6.330) | Student's *t*-test | *t* = 1.805 | = 0.088 |
| **5MSOP** | — | 0.184 ± 0.053  (0.176) | — | 0.197 ± 0.043  (0.213) | Student's *t*-test | *t* = -0.633 | = 0.535 |
| **7MSOH** | — | 0.073 ± 0.027  (0.064) b | — | 0.145 ± 0.064  (0.122) a | Mann-Whitney *U* test | *U* = 12.000 | = 0.005 |
| **8MSOO** | — | 0.684 ± 0.079  (0.658) | — | 0.865 ± 0.314  (0.814) | Mann-Whitney *U* test | *U* = 32.000 | = 0.186 |
| **I3M** | 0.041 ± 0.034  (0.032) c | 1.177 ± 0.350  (1.194) b | 4.076 ± 1.060  (3.979) a | 0.030 ± 0.023  (0.024) c | Generalized least squares | *LR* = 54.850 | < 0.001 |
| **4MOI3M** | 0.025 ± 0.017  (0.022) c | 0.046 ± 0.033  (0.037) b | 0.296 ± 0.129  (0.282) a | 0.008 ± 0.013  (0.000) d | Generalized least squares^2^ | *LR* = 49.157 | < 0.001 |
| **1MOI3M** | — | 0.008 ± 0.009  (0.005) b | 0.049 ± 0.017  (0.050) a | — | Student's *t*-test | *t* = -6.677 | < 0.001 |
| **Total** | 34.668 ± 6.611  (34.915) | 34.889 ± 1.994  (34.637) | 30.847 ± 4.315  (30.718) | 34.131 ± 5.136  (33.858) | Generalized least squares | *LR* = 6.309 | = 0.098 |

^1^Data were square-root transformed prior to analysis. ^2^Data were log transformed prior to analysis. GLS concentrations labeled with different letters are significantly different (*P* < 0.05). LR, likelihood ratio; —, not detected; 3But, 3-butenyl; for other abbreviations refer to the legend of Table S1

**Table S3** GLS amounts of newly emerged *P. armoraciae* adults and after five days of feeding on three different *Arabidospis* lines

| **GLS side chain** | **nmol sequestered GLS per adult (mean ± SD (median); *N* = 10)** | | | | **Statistical method** | **Statistics** | ***P* value** |
| --- | --- | --- | --- | --- | --- | --- | --- |
|  | **Newly emerged beetles** | **Beetles fed**  **on wild type** | **Beetles fed**  **on *myb*** | **Beetles fed**  **on *cyp*** |  |  |  |
| **Allyl** | 65.060 ± 10.849  (66.440) a | 41.818 ± 7.758  (40.890) c | 56.437 ± 12.800  (55.330) ab | 45.775 ± 10.630  (47.865) bc | ANOVA | *F* = 9.746 | < 0.001 |
| **3But** | 0.947 ± 0.515  (0.884) b | 1.597 ± 0.635  (1.575) a | 0.193 ± 0.162  (0.120) c | 1.332 ± 0.515  (1.155) ab | ANOVA^1^ | *F* = 25.891 | < 0.001 |
| **3MSOP** | — | 2.371 ± 0.417  (2.404) a | — | 1.819 ± 0.400  (1.762) b | Student's *t*-test | *t* = 3.021 | = 0.007 |
| **4MSOB** | — | 7.366 ± 1.925  (6.943) | — | 6.299 ± 1.627  (6.729) | Student's *t*-test | *t* = 1.340 | = 0.197 |
| **4MTB** | — | 15.738 ± 3.466  (16.099) | — | 13.973 ± 3.293  (13.954) | Student's *t*-test | *t* = 1.167 | = 0.258 |
| **5MSOP** | — | 0.381 ± 0.108  (0.367) | — | 0.415 ± 0.095  (0.426) | Student's *t*-test | *t* = -0.738 | = 0.470 |
| **7MSOH** | — | 0.150 ± 0.048  (0.146) b | — | 0.301 ± 0.120  (0.283) a | Mann-Whitney *U* test | *U* = 6.000 | = 0.001 |
| **8MSOO** | — | 1.438 ± 0.248  (1.397) | — | 1.798 ± 0.578  (1.744) | Mann-Whitney *U* test | *U* = 30.000 | = 0.140 |
| **I3M** | 0.084 ± 0.080  (0.060) c | 2.476 ± 0.822  (2.574) b | 8.754 ± 2.423  (9.534) a | 0.061 ± 0.047  (0.053) c | Generalized least squares | *LR* = 51.434 | < 0.001 |
| **4MOI3M** | 0.048 ± 0.033  (0.044) b | 0.093 ± 0.056  (0.078) b | 0.626 ± 0.263  (0.584) a | 0.016 ± 0.027  (0.000) c | ANOVA^2^ | *F* = 54.521 | < 0.001 |
| **1MOI3M** | — | 0.017 ± 0.021  (0.009) b | 0.106 ± 0.040  (0.108) a | — | Mann-Whitney *U* test | *U* = 1.500 | < 0.001 |
| **Total** | 66.139 ± 11.220  (67.185) | 73.446 ± 11.438  (71.724) | 66.116 ± 12.010  (66.353) | 71.788 ± 11.063  (71.739) | ANOVA | *F* = 1.108 | = 0.359 |

^1^Data were square-root transformed prior to analysis. ^2^Data were log transformed prior to analysis. GLS amounts labeled with different letters are significantly different (*P* < 0.05). LR, likelihood ratio; —, not detected. For GLS and host plant abbreviations, refer to legend of Table S1 and Table S2.

**Table S4** GLS amounts detected in *P. armoraciae* feces after feeding on different *Arabidopsis* lines for five days

| **GLS side chain** | **nmol excreted GLS per beetle (mean ± SD (median); *N* = 10)** | | | **Statistical method** | **Statistics** | ***P* value** |
| --- | --- | --- | --- | --- | --- | --- |
|  | **wild type** | ***myb*** | ***cyp*** |  |  |  |
| **Allyl** | 9.946 ± 3.364  (9.896) a | 0.879 ± 0.873 (0.648) b | 7.601 ± 3.069 (7.475) a | ANOVA^2^ | *F* = 54.564 | < 0.001 |
| **3MSOP** | 1.737 ± 0.406  (1.833) a | — | 0.748 ± 0.330 (0.809) b | Student's *t*-test | *t* = 5.983 | < 0.001 |
| **4MSOB** | 13.456 ± 2.961  (13.967) a | — | 5.341 ± 1.986 (5.768) b | Student's *t*-test | *t* = 7.197 | < 0.001 |
| **5MSOP** | 1.172 ± 0.264  (1.083) a | — | 0.823 ± 0.324 (0.797) b | Student's *t*-test | *t* = 2.638 | = 0.017 |
| **7MSOH** | 0.409 ± 0.085  (0.382) a | — | 0.242 ± 0.088 (0.245) b | Student's *t*-test | *t* = 4.325 | < 0.001 |
| **8MSOO** | 0.912 ± 0.234  (0.958) a | — | 0.539 ± 0.232 (0.537) b | Student's *t*-test | *t* = 3.582 | = 0.002 |
| **I3M** | 2.632 ± 0.709  (2.545) a | 0.741 ± 0.335 (0.700) b | *0.000 ± 0.000* (*0.000*)*^1^* | Student's *t*-test | *t* = 7.629 | < 0.001 |
| **4MOI3M** | 0.525 ± 0.101  (0.527) | 0.498 ± 0.147 (0.480) | *0.001 ± 0.004* (0.000)*^1^* | Student's *t*-test | *t* = 0.477 | = 0.639 |
| **Total** | 30.789 ± 7.344  (33.095) a | 2.118 ± 0.902 (2.068) c | 15.295 ± 4.851 (13.631) b | ANOVA^3^ | *F* = 136.860 | < 0.001 |

—, not detected. ^1^If a GLS was only detected in one sample, the mean is written in italics and data was not included in the statistical analysis. ^2^Data were square-root transformed prior to analysis. ^3^Data were log transformed prior to analysis. GLS amounts labeled with different letters are significantly different (*P* < 0.05). For GLS and food plant abbreviations, refer to legend of Table S1 and S2.

**Supplementary Figures**


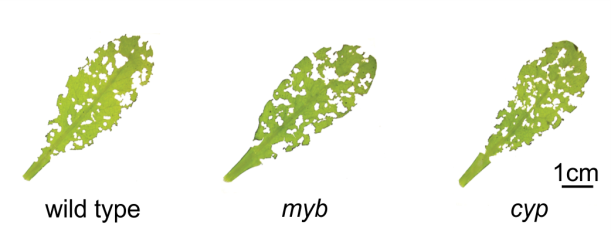


**Fig. S1** Representative feeding patterns of five *P. armoraciae* adults on *Arabidopsis* wild type, *myb28myb29* (*myb*), and *cyp79b2cyp79b3* (*cyp*), respectively, for one day.


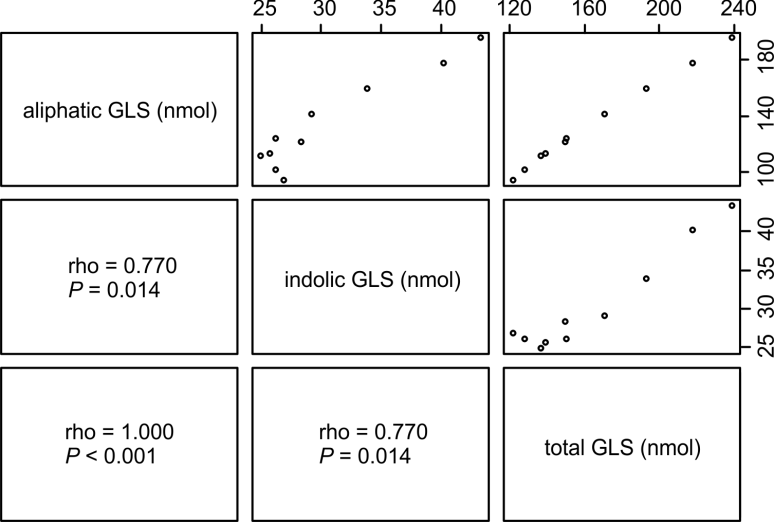


**Fig. S2** Spearman’s rank correlation matrix between the ingested aliphatic GLS, indolic GLS and total GLS amounts by *P. armoraciae* adults from *Arabidopsis* wild type in five days. The correlation coefficients and significance, and scatterplot matrices are show on the left and right side of the diagonal, respectively. Aliphatic GLS: sum of 3MSOP GLS, 4MSOB GLS, 4MTB GLS, 5MSOP GLS, 7MSOH GLS, 8MSOO GLS; indolic GLS: sum of I3M GLS, 1MOI3M GLS, 4MOI3M GLS. For GLS abbreviations, refer to legend of Table S1.

**Supplementary Information and Figure on 4-methylsulfinylbutyl GLS metabolism in the cabbage stem flea beetle, *Psylliodes chrysoecphala***

*Metabolism of 4-methylsulfinylbutyl GLS in the cabbage stem flea beetle Psylliodes chrysocephala* To determine whether the cabbage stem flea beetle, *Psylliodes chrysocephala* (L.) (Chrysomelidae), metabolizes sequestered 4-methylsulfinylbutyl (4MSOB) GLS to 4-methylthiobutyl- (4MTB) and 3-butenyl (3But) GLS, we performed a feeding experiment with newly emerged beetles. *P. chrysocephala* adults were reared on three to four-week-old potted *Brassica rapa* cv. Yu Tsai Sum plants (Known-You Seed Co. Ltd., Kaohsiung, China) as described in Beran et al. (2018). Newly emerged adults were placed dorsally onto sticky tape and forced to drink 0.2 µL of an aqueous solution containing 10 nmol of 4MSOB GLS (purchased from Phytoplan, Heidelberg, Germany) using a pipette. Beetles fed with pure water served as a control. To allow the metabolism of ingested and sequestered 4MSOB GLS, fed beetles were kept in Petri dishes with detached leaves of *B. rapa* for three days. Afterwards, adults were frozen in liquid nitrogen and stored at -20°C until GLS analysis. There were four and six biological replicates for the control and 4MSOB GLS treatments, respectively, each consisting of five adults.

We detected a minor amount of 4MTB GLS as a contaminant in the 4MSOB GLS solution that was fed to *P. chrysocephala*. However, the 4MTB GLS amount detected in *P. chrysocephala* beetles was significantly lower than that detected in the fed 4MSOB GLS solution (Fig. S3; Student's *t*-test, 4MTB GLS: *t* = 37.269, *P* < 0.001), which indicates that *P. chrysocephala* does not metabolize significant quantities of sequestered 4MSOB GLS to 4MTB GLS. 3But GLS is known to be present in *P. chrysocephala* beetles reared on *B. rapa* (Beran et al. 2018). In our experiment, we found no difference between the 3But GLS levels in control beetles and 4MSOB GLS-fed beetles (Fig. S3; Student's *t*-test, 3But GLS: *t* = 0.919, *P* = 0.385), making it unlikely that *P. chrysocephala* adults convert 4MSOB GLS to 3But GLS.

**
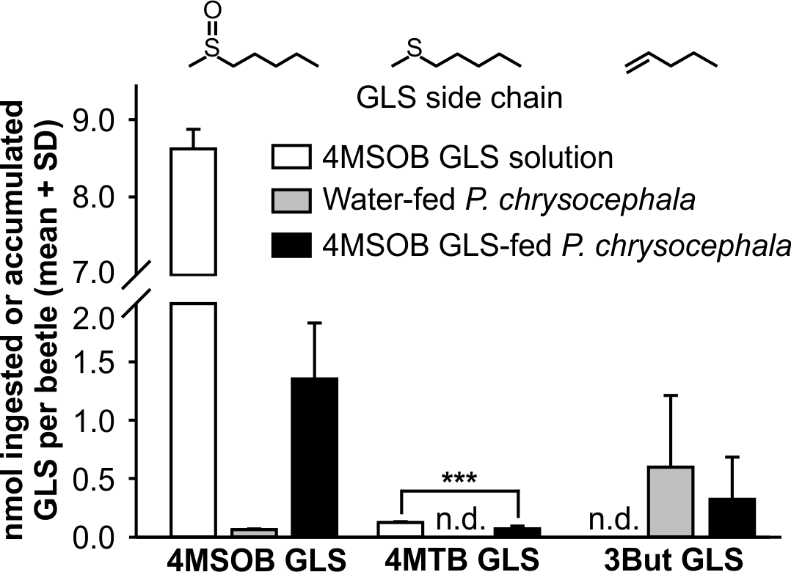
**

**Fig. S3** *Psylliodes chrysocephala* adults do not metabolize sequestered 4-methylsulfinylbutyl (4MSOB) GLS. Adults were fed with an aqueous 4MSOB GLS solution or water as a control, and harvested for GLS extraction after three days feeding on *Brassica rapa* leaves (*N* = 4 – 6). The 4MSOB GLS solution fed to adults contained a small amount of 4-methylthiobutyl (4MTB) GLS as a contaminant. The 4MTB GLS amount in the fed 4MSOB GLS solution was compared with the 4MTB GLS amount detected in *P. chrysocephala* by Student’s *t*-test. 3-Butenyl (3But) GLS was not detected in the 4MSOB GLS solution, but was detected in control beetles and fed beetles. 3But GLS amounts in adults were log-transformed and compared by Student’s *t*-test. n.d., not detected, ****P* < 0.001.

**Reference**

Beran F, Sporer T, Paetz C, Ahn S-J, Betzin F, Kunert G, Shekhov A, Vassão DG, Bartram S, Lorenz S, Reichelt M (2018) One pathway is not enough: the cabbage stem flea beetle *Psylliodes chrysocephala* uses multiple strategies to overcome the glucosinolate-myrosinase defense in its host plants. Front Plant Sci 9:1754. https://doi.org/10.3389/fpls.2018.01754
